# Susceptibility to *L. sigmodontis* infection is highest in animals lacking IL-4R/IL-5 compared to single knockouts of IL-4R, IL-5 or eosinophils

**DOI:** 10.1186/s13071-019-3502-z

**Published:** 2019-05-20

**Authors:** Stefan J. Frohberger, Jesuthas Ajendra, Jayagopi Surendar, Wiebke Stamminger, Alexandra Ehrens, Benedikt C. Buerfent, Katrin Gentil, Achim Hoerauf, Marc P. Hübner

**Affiliations:** 1 Institute for Medical Microbiology, Immunology and Parasitology, University Hospital of Bonn, Bonn, Germany; 2 Center for Human Genetics, University Hospital of Marburg, Marburg, Germany; 3 Institute of Medical Microbiology, Justus Liebig University Giessen, Giessen, Germany; 4 German Centre for Infection Research (DZIF), Partner Site Bonn-Cologne, Bonn, Germany

**Keywords:** *Litomosoides sigmodontis*, Filariae, Eosinophil, IL-4, IL-5, Microfilaria, Embryogenesis, Macrophage

## Abstract

**Background:**

Mice are susceptible to infections with the rodent filarial nematode *Litomosoides sigmodontis* and develop immune responses that resemble those of human filarial infections. Thus, the *L. sigmodontis* model is used to study filarial immunomodulation, protective immune responses against filariae and to screen drug candidates for human filarial diseases. While previous studies showed that type 2 immune responses are protective against *L. sigmodontis*, the present study directly compared the impact of eosinophils, IL-5, and the IL-4R on the outcome of *L. sigmodontis* infection.

**Methods:**

Susceptible wildtype (WT) BALB/c mice, BALB/c mice lacking eosinophils (dblGATA mice), IL-5^−/−^ mice, IL-4R^−/−^ mice and IL-4R^−/−^/IL-5^−/−^ mice were infected with *L. sigmodontis*. Analyses were performed during the peak of microfilaremia in WT animals (71 dpi) as well as after IL-4R^−/−^/IL-5^−/−^ mice showed a decline in microfilaremia (119 dpi) and included adult worm counts, peripheral blood microfilariae levels, cytokine production from thoracic cavity lavage, the site of adult worm residence, and quantification of major immune cell types within the thoracic cavity and spleen.

**Results:**

Our study reveals that thoracic cavity eosinophil numbers correlated negatively with the adult worm burden, whereas correlations of alternatively activated macrophage (AAM) numbers with the adult worm burden (positive correlation) were likely attributed to the accompanied changes in eosinophil numbers. IL-4R^−/−^/IL-5^−/−^ mice exhibited an enhanced embryogenesis achieving the highest microfilaremia with all animals becoming microfilariae positive and had an increased adult worm burden combined with a prolonged adult worm survival.

**Conclusions:**

These data indicate that mice deficient for IL-4R^−/−^/IL-5^−/−^ have the highest susceptibility for *L. sigmodontis* infection, which resulted in an earlier onset of microfilaremia, development of microfilaremia in all animals with highest microfilariae loads, and an extended adult worm survival.

**Electronic supplementary material:**

The online version of this article (10.1186/s13071-019-3502-z) contains supplementary material, which is available to authorized users.

## Background

Parasitic filarial nematodes can cause debilitating diseases that stigmatize the affected individuals by causing blindness and severe dermatitis in onchocerciasis patients and lymphedema in limbs (elephantiasis) and scrotum (hydrocele) in lymphatic filariasis patients. Due to the chronic nature of these diseases and the inability of the affected patients to work, onchocerciasis and lymphatic filariasis present a huge socio-economic problem [1, 2]. From human filarial infections it is known that patients develop type 2 immune responses, which are characterized by an eosinophilia, increased production of type 2 cytokines such as IL-4, IL-5, eosinophil-associated molecules [3], and increased numbers of innate lymphocyte cells [4] and alternatively activated macrophages [5]. Furthermore, regulatory immune responses develop during human filarial infection that suppress both type 1 and type 2 immune responses [6, 7]. Interestingly, these type 2 immune responses are associated with protective immune responses and the development of filarial pathology during onchocerciasis, as patients that develop hyperreactive onchocerciasis with severe skin disease have the strongest type 2 immune responses, but have reduced microfilariae (MF) levels [8, 9]. Similarly, in lymphatic filariasis, only ~50% of patients develop microfilaremia, and those patients have been shown to have increased adaptive immune responses and higher parasite-specific IL-5 levels [10]. Development of lymphedema on the other hand has been associated with pronounced parasite-specific Th1 and Th17 responses [11].

In order to obtain a better understanding of protective immune responses during filariasis and based on the resistance of immunocompetent laboratory mice to human pathogenic filariae, the *Litomosoides sigmodontis* mouse model was developed. BALB/c mice are fully susceptible to *L. sigmodontis* infection and the nematode can undergo its full life-cycle under laboratory conditions [12, 13]. *Litomosoides sigmodontis*-infected mice develop immune responses that resemble those of human filarial infections and previous studies using *L. sigmodontis*-infected mice helped us to obtain a better understanding of the filarial immunomodulation and protective immune responses involved. Thus, *L. sigmodontis* infection was shown to provide a beneficial impact on allergic sensitization in asthma [14], type III hypersensitivity [15], modulate vaccine [16, 17] and T cell responses [18–20], and to induce AAM [21], regulatory T cells [22] as well as type 2 innate lymphoid cells (ILC2s) [23]. With regards to protection, besides type 2 immunity, a variety of immune responses including type 1 immune responses and innate cell types were identified as essential, whereas the induction of regulatory responses favours parasite survival [22, 24–31]. For type 2 immune responses, eosinophils as well as type 2 cytokines have been previously shown as essential for protection against *L. sigmodontis*. Thus, mice on a semi-resistant 129/SvJ background have an increased *L. sigmodontis* worm burden in the absence of the eosinophil products eosinophil peroxidase (EPO) and major basic protein (MBP) [32]. Similarly, eotaxin1-deficient mice had an increased *L. sigmodontis* adult worm burden [33]. Lack of the type 2 cytokine IL-5, which is also essential for eosinophil generation and survival, was previously shown to impair adult worm clearance during *L. sigmodontis* infection [34–37]. Furthermore, IL-4 is essentially involved in protective immune responses against *L. sigmodontis*, as semi-resistant C57BL/6 mice developed patent infections in the absence of IL-4 [38] and susceptible IL-4 deficient-BALB/c mice had significantly increased MF levels compared to the respective wildtype (WT) controls [36, 39]. IL-4 and IL-13 signal *via* the IL-4 receptor (IL-4R), which is essential for the development of alternatively activated macrophages (AAM). AAM were previously shown to expand within the thoracic cavity of *L. sigmodontis-*infected mice [21]. According to the protective mechanisms described above for IL-4 and IL-5, BALB/c mice lacking both the IL-4R and IL-5 had a significantly increased *L. sigmodontis* adult worm burden and microfilaremia in comparison to WT controls [40].

The aim of the present study was to directly compare the protective role of different components of the type 2 immune response during filarial infection. Therefore, we compared *L. sigmodontis* infection in BALB/c WT mice with BALB/c mice lacking eosinophils (dblGATA) and BALB/c mice deficient in either IL-4R, IL-5 or both IL-4R/IL-5 during the peak of microfilaremia in WT mice (71 days post-infection, dpi) and a late time point of infection, where the infection is cleared in the majority of WT animals and IL-4R^−/−^/IL-5^−/−^ started to show a decline in the peripheral blood MF counts (119 dpi). At both time points adult worm burden was increased in dblGATA, IL-5^−/−^, and IL-4R^−/−^/IL-5^−/−^ mice compared to WT controls, indicating the essential contribution of eosinophils in adult worm clearance. Microfilaremia occurred in all immunodeficient animals at an earlier time point than in WT controls, with all dblGATA, IL-4R^−/−^ and IL-4R^−/−^/IL-5^−/−^ mice but only 50% of WT controls and 70% of IL-5^−/−^ mice developing microfilaremia, respectively. MF load was highest in IL-4R^−/−^/IL-5^−/−^ mice, followed by dblGATA and IL-5^−/−^ mice, and persisted in those mice for > 120 dpi, while MF declined in IL-4R^−/−^ and WT controls following 78 dpi. None of the measured cytokines within the thoracic cavity (IL-4, IL-5, IL-13, IFNγ) correlated with the adult worm burden or microfilaremia at 71 or 119 dpi. Thoracic cavity eosinophil numbers correlated negatively with the adult worm at 71 dpi, whereas AAMs showed a positive correlation with the adult worm burden at 119 dpi and a negligible negative correlation with the MF load, which was probably attributed to the associated changes in eosinophils. Neutrophil numbers in the spleen further correlated positively with the adult worm burden and MF load at the later time point.

## Methods

### Mice and infection

All animals were bred at the animal facilities of the University Hospital of Bonn (House for Experimental Therapy) and housed during the experiment at the animal facility of the Institute for Medical Microbiology, Immunology and Parasitology. Mice were kept in individually ventilated cages with access to food and water *ad libitum.*

BALB/c WT and IL-4R^−/−^ mice (BALB/c-Il4ratm1Sz/J) were purchased from Janvier (Le Genest-St.-Isle, France) and Charles River (Erkrath, Germany), respectively. dblGATA mice were originally obtained from The Jackson Laboratory (Bar Harbor, ME, USA), IL-5^−/−^ and IL-4R^−/−^/IL-5^−/−^ from Prof. Dr. Klaus Matthaei (Matthaei, Stem Cell & Gene Targeting Laboratory, ANU College of Medicine, Biology and Environment, Canberra, Australia). Thus, mice were used that lack signalling *via* IL-4R, which responds to IL-4 as well as IL-13, lack eosinophils (dblGATA and IL-5^−/−^) or lack both IL-4R/IL-5.

Age and sex-matched mice were infected at 6–8 weeks of age with *L. sigmodontis via* natural infection with the intermediate host as previously described [36]. To ensure equal infection of all groups, mice were exposed to the same batch of *Ornithonyssus bacoti* mites containing infective *L. sigmodontis* L3 larvae. Necropsies were performed at 71 and 119 dpi. Infection of mice was confirmed by screening for adult worms in the thoracic cavity and peritoneum as well as microfilariae in the peripheral blood.

### Parasite recovery

Mice were euthanized with an overdose of isoflurane (Abbvie, Wiesbaden, Germany) during the peak of microfilaremia in WT animals at 71 dpi and at the time MF started to decline in the IL-4R^−/−^/IL-5^−/−^ animals and when the majority of adult worms are cleared from the WT animals, 119 dpi. The adult worm burden within the thoracic cavity and the peritoneum was quantified and gender and lengths of the filariae were determined. Peripheral blood was taken weekly from the facial vein for MF counts, from 49 to 119 dpi. Fifty microlitres of peripheral blood was added to 1 ml of red blood cell (RBC) lyses buffer (Thermo Fisher Scientific, Waltham, MA, USA) and incubated for 10 min at room temperature. Afterwards the samples were centrifuged at 400×*g* for 5 min. MF were counted from the whole pellet using a microscope at 10× magnification.

### Analysis of female worm embryogenesis

Embryonic stages (egg, morula, pretzel, stretched MF) were determined and enumerated from two female worms per animal (total of 10 worms per group) isolated from WT, dblGATA, IL-5^−/−^ and IL-4R^−/−^/IL-5^−/−^ mice at 71 dpi. Worms were individually homogenized by using a mortar in 80 µl of phosphate-buffered saline (PBS) and 20 µl of Hinkelmann solution (0.5% eosin Y, 0.5% phenol, 0.185% formaldehyde in distilled water). Embryonic stages in 10 µl were determined and counted under a light microscope (10× magnification).

### Isolation of thoracic cavity and spleen cells

Pleural lavages with RPMI 1640 media (PAA) were performed at necropsy to acquire thoracic cavity cells. The first mililitre was collected, cells were separated by centrifugation at 400×*g* for 5 min and the supernatant was stored at − 20 °C for subsequent cytokine measurements. Isolated cells from the first lavage were combined with cells gathered during a second lavage with 4 ml of RPMI 1640 media. Spleens were isolated and single cell suspensions were prepared as previously described [41].

### Measurement of cytokines by ELISA

Cytokine measurements were performed within the first ml of thoracic cavity lavage by ELISA. IL-4, IL-5, IL-13 and IFNγ (all Thermo Fisher Scientific) were all measured according to the manufacturer’s protocol.

### Flow cytometric analyses of thoracic cavity and spleen cells

Thoracic cavity and spleen cells were analyzed by flow cytometry. Cells were blocked with PBS/1% BSA including 0.1 % rat IgG (Sigma-Aldrich, St. Louis, MO, USA) and stained. For intracellular staining, cells were fixed overnight in fixation/permeabilization buffer (Thermo Fisher Scientific). The next day cells were washed with PBS and centrifuged at 400×*g* for 5 min at 4 °C. The supernatant was discarded, and cells were permeabilized with Perm buffer (Thermo Fisher Scientific) for 20 min at room temperature.

Flow cytometric analysis was performed using a combination of the following surface markers: CD4 FITC, CD8 APC, SiglecF PE, F4/80 PerCP Cy5.5 and Gr1 Pe-Cy7. CD4^+^ T and CD8^+^ T cells were identified as CD4^high^ or CD8^high^ cells, respectively; neutrophils as Gr1^high^, SiglecF^low^; eosinophils as SiglecF^high^, F4/80^low^; macrophage populations were identified as F4/80^high^, SiglecF^low^ and alternatively activated macrophages as F4/80^high^, SiglecF^low^, RELMα^high^. All antibodies except for RELMα were obtained from Thermo Fisher Scientific. Intracellular staining for RELMα was performed utilizing a two-step staining protocol using rabbit anti-mouse RELMα (PeproTech, Hamburg, Germany) followed by a goat anti-rabbit Alexa Fluor 488 conjugated antibody (Invitrogen, Carlsbad, CA, USA). CD4 FITC and goat anti-rabbit Alexa Fluor 488 were used in separated panels. The gating strategy to identify the different cell populations is shown as Additional file 1: Figure S1. Flow cytometry was performed using a BD FACS Canto system and data was subsequently analyzed using the FACS Diva 5.1 software (BD Biosciences, Heidelberg, Germany). During analysis, cut-offs were set using the fluorescence minus one approach.

### Statistics

Statistical analyses were performed with GraphPad Prism software v.5.03 (GraphPad Software, San Diego, CA, USA). Normal distribution of the data was tested with D’agostino test. Parametrically distributed data were analyzed by one-way ANOVA followed by Dunnett’s test, whereas non-parametrically distributed data and data of non-sufficient animal numbers for parametric testing were analyzed by Kruskal-Wallis test followed by Dunn’s *post-hoc* test. *P*-values of< 0.05 were considered statistically significant. Data from pooled experiments were tested for homoscedasticity by two-way ANOVA and Spearman’s test for heteroscedasticity using GraphPad Prism software v.8. Only experiments that did not pass the heteroscedasticity test were pooled.

## Results

### IL-4R, IL-5 and eosinophils control the occurrence of microfilaremia, whereas IL-5 and eosinophils impair adult worm survival and maintenance of microfilaremia

**Fig. 1:**
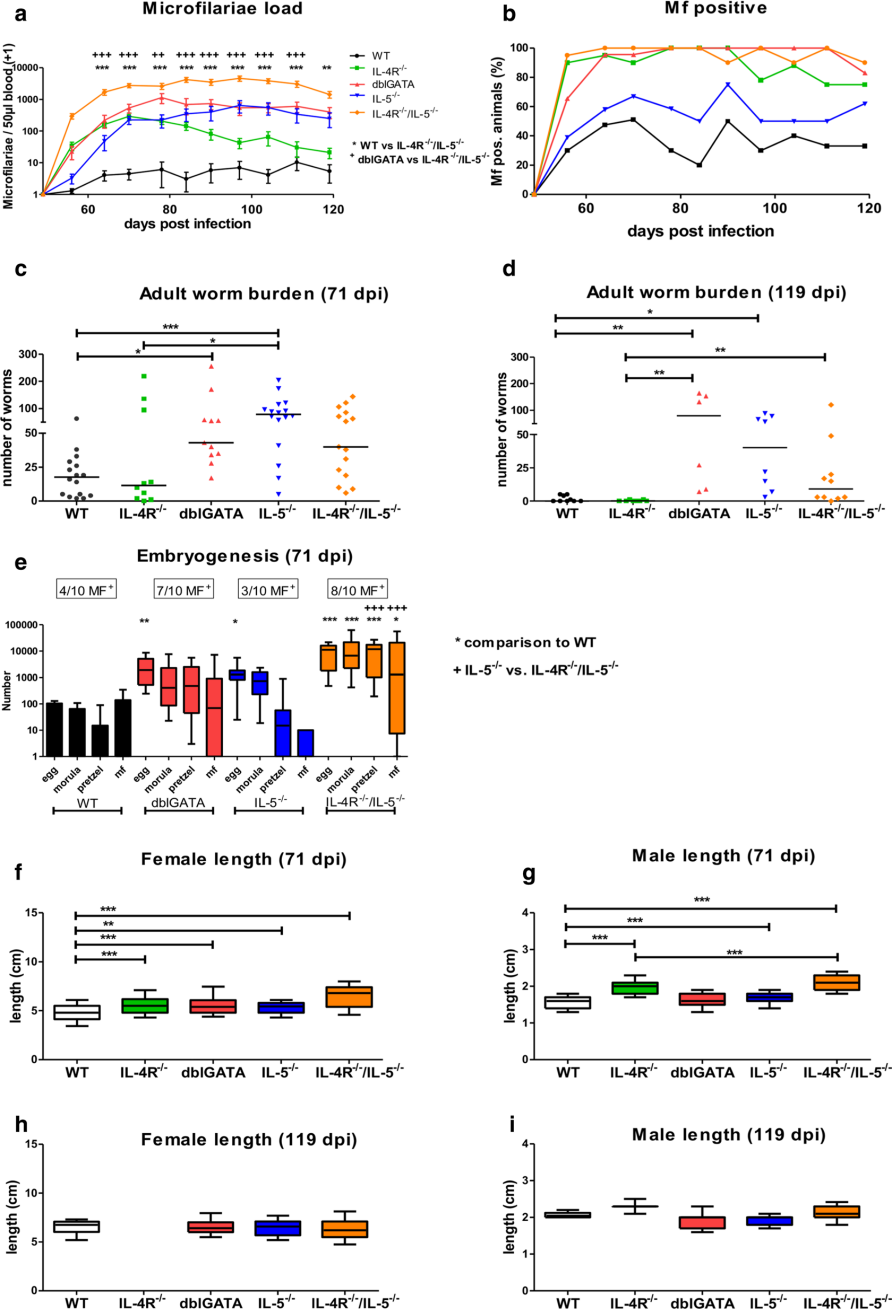
IL-4R and IL-5/eosinophils control microfilaremia, whereas IL-5 and eosinophils impair adult worm survival and maintenance of microfilaremia. **a** Microfilariae count per 50 μl of peripheral blood throughout *L. sigmodontis* infection and **b** frequency of wildtype (WT) controls, IL-4R^**−/−**^, dblGATA, IL-5^**−/−**^ and IL-4R^**−/−**^/IL-5^**−/−**^ mice that develop microfilaremia. Adult worm burden (**c**, **d**), embryogenesis of female adult worms staged as eggs, morulae, pretzel and stretched microfilariae (mf) at 71 days post-infection (dpi) with numbers of female worms containing stretched microfilariae within their uteri indicated above the different mouse strains (**e**), and female (**f**, **h**) and male worm length (**g**, **i**) at 71 (**c**, **f**, **g**) and 119 (**d**, **h**, **i**) dpi. Results are shown as means ± SEM (**a**, **b**), medians (**c**, **d**), and box and whisker plots with 10th and 90th percentiles (**e**–**i**). Data were analyzed using two-way ANOVA followed by Bonferroni’s *post-hoc* test (**a**), one-way ANOVA followed by the Dunnett’s test (**f**) and Kruskal–Wallis test followed by Dunn’s multiple comparison test (**c**–**e**, **g**–**i**). *P < 0.05, **P < 0.01, ***P < 0.001. Data shown in **a–c** are pooled from two independent experiments at 71 dpi with a total of 10–16 mice per group. Data shown in **d**, **f**–**i** are from one experiment with 6–10 mice per group and data shown in **e** are from a single experiment with 5 mice per group and analysis of 2 female worms per mouse

In order to directly compare the impact of IL-4R, IL-5, IL-4R/IL-5 and eosinophils on the development of *L. sigmodontis* infection, we analyzed the MF burden over time, the frequency of animals developing microfilaremia and determined total adult worm numbers and worm lengths at 71 dpi, which represents a time point around the microfilariae peak in WT animals, and at 119 dpi, a time point most WT animals cleared the infection and IL-4R^−/−^/IL-5^−/−^mice showed a first decline in the MF load. Immunodeficient mice (IL-4R^−/−^, IL-5^−/−^, IL-4R^−/−^/IL-5^−/−^, dblGATA BALB/c mice) exhibited increased numbers of peripheral MF throughout the infection compared to WT controls (Fig. 1a). Interestingly, in all immunodeficient mice tested, the release of MF into the peripheral blood occurred earlier than in WT controls, with IL-4R^−/−^/IL-5^−/−^ having the highest MF counts, which was significantly increased in comparison to WT and dblGATA mice. Ninety percent of IL-4R and 95% of IL-4R/IL-5 deficient mice had peripheral microfilaremia at 56 dpi, whereas microfilaremia was present in 39% of IL-5 deficient and 66% of dblGATA mice and 30% of the WT controls at that time point (Fig. 1b). The peak of microfilaremia was observed in WT and dblGATA mice at 78 dpi (~ 746 MF/50 µl blood), in IL-4R^−/−^ (~ 294 MF/50 µl blood) at 70 dpi, and in IL-5^−/−^ (~ 639 MF/50 µl blood) and IL-4R^−/−^/IL-5^−/−^ at 97 dpi (~ 4600 MF/50 µl blood) (Fig. 1a). Microfilaremia persisted in IL-4R^−/−^/IL-5^−/−^, dblGATA, and IL-5^−/−^ mice for > 120 dpi, while microfilaremia declined in IL-4R^−/−^ and WT controls following 78 dpi (Fig. 1a). The frequency of MF-positive animals was considerably higher in all immunodeficient mice (dblGATA, IL-4R^−/−^/IL-5^−/−^, IL-4R^−/−^ mice: 100 %, IL-5^−/−^ mice: 75%) compared to WT controls (50%; Fig. 1b). Adult worm counts were increased in mice deficient for dblGATA, IL-5 as well as IL-4R/IL-5 compared to WT and IL-4R deficient mice, reaching statistical significance for the comparison of dblGATA and IL-5^−/−^ with WT mice on 71 dpi and dblGATA as well as IL-5^−/−^ mice in comparison to WT mice at 119 dpi (Fig. 1c, d). At 119 dpi an increased number of granuloma was observed in the IL-4R^−/−^/IL-5^−/−^ mice, which hampered the exact worm counts and may explain the lower worm numbers in comparison to the dblGATA and IL-5^−/−^ mice. Additional experiments with dblGATA and WT animals confirmed the increased susceptibility of dblGATA mice (Additional file 2: Table S1), revealing a significantly increased MF load at 76 dpi and an increased adult worm burden at 60 and 90 dpi in comparison to WT controls. However, only 70% of the dblGATA animals developed microfilaremia, which may be due to the lower adult worm burden in this experiment in comparison to the data shown in Fig. 1.

In order to determine whether the increased MF counts in IL-4R^−/−^/IL-5^−/−^, dblGATA and IL-5^−/−^ mice was due to an enhanced embryogenesis of female adult worms and therefore, MF release in comparison to WT controls, embryograms were performed. Embryograms from 71 dpi demonstrated that female adult worms from IL-4R^−/−^/IL-5^−/−^ animals had significantly higher numbers of all embryonal stages (eggs, morulae, pretzel and stretched MF) compared to WT animals and of the later embryonal stages (pretzel and stretched MF) in comparison to IL-5^−/−^ mice (Fig. 1e). In contrast, female adult worms from dblGATA and IL-5^−/−^ mice had increased numbers of the early embryonal stages (Kruskal–Wallis H-test: *χ*^2^ = 94.33, *df* = 16, *P* = 0.0001 followed by Dunn’s *post-hoc* test; eggs: *P *< 0.01/*P *< 0.05; morulae: *P* > 0.05), but the number of stretched MF was not significantly increased. These data indicate that the highest MF count in IL-4R^−/−^/IL-5^−/−^ animals is, in part, due to an enhanced embryogenesis.

Female and male filariae isolated from all tested immunocompromised mice were significantly longer compared to filariae from WT controls at 71 dpi (mean female worm length: WT, 4.82 cm; IL-4R^−/−^, 5.54 cm; dblGATA, 5.62 cm; IL-5^−/−^, 5.30 cm; IL-4R^−/−^/IL-5^−/−^, 6.51 cm; mean male worm length: WT, 1.54 cm; IL-4R^−/−^, 1.97 cm; dblGATA, 1.62 cm; IL-5^−/−^, 1.68 cm; IL-4R^−/−^/IL-5^−/−^, 2.11 cm; Fig. 1f, g). Differences in female and male worm lengths were not observed between the different mouse strains at 119 dpi (Fig. 1h, i). Furthermore, the ratio of male and female adult worms at 71 and 119 dpi was not altered in any of the mouse strains tested (data not shown).

These results indicate that occurrence of microfilaremia is controlled by the IL-4R and IL-5/eosinophils, while maintenance of microfilaremia seems to be mainly controlled by IL-5/eosinophils. This effect on microfilaremia was further promoted by the combined deficiency of IL-4R and IL-5.

### Negative association of thoracic cavity eosinophils with adult worm survival and of AAM with microfilaremia

**Fig. 2:**
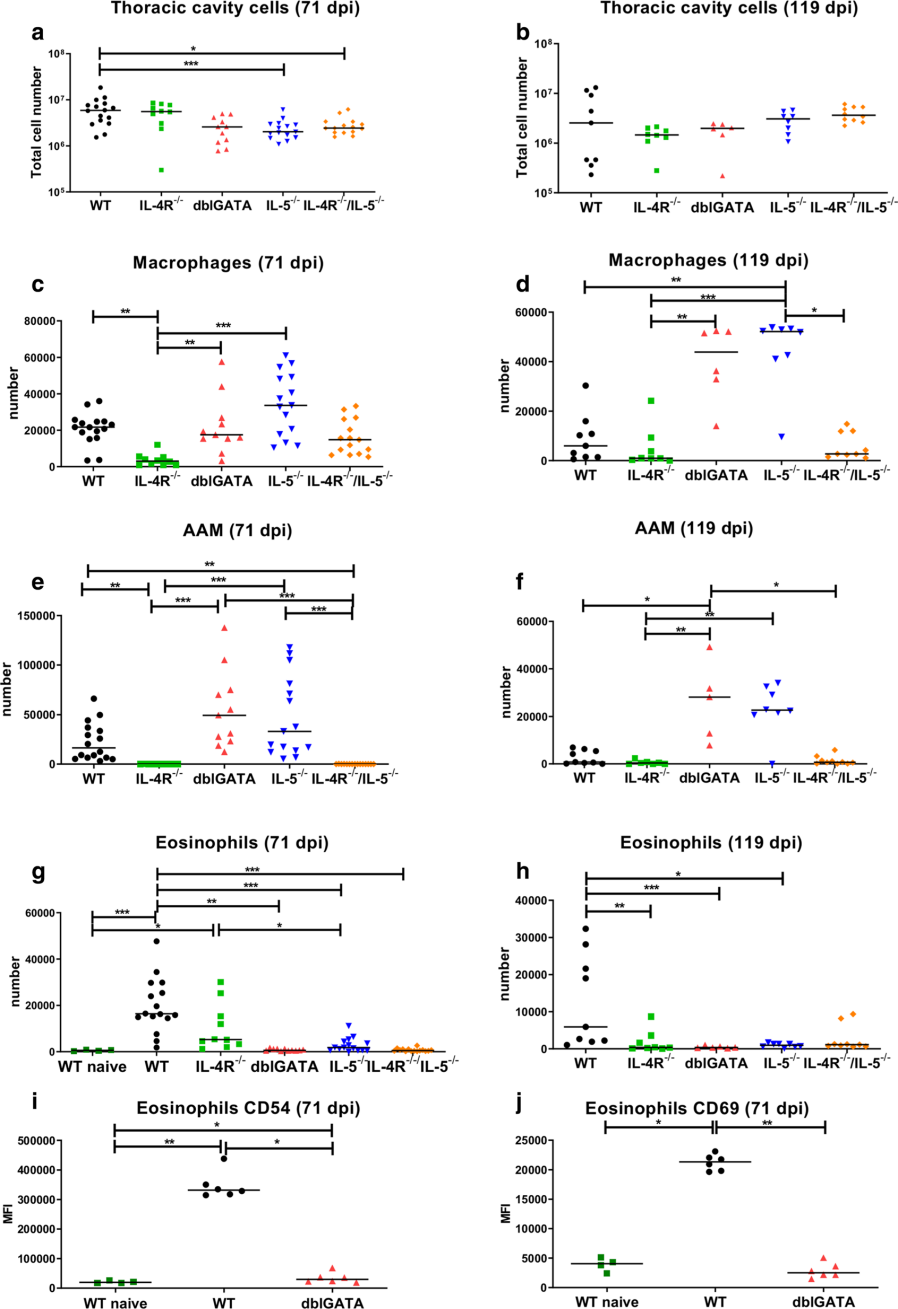
Thoracic cavity cells of *L. sigmodontis*-infected IL-5^−/−^, dblGATA and IL-4R^−/−^/IL-5^−/−^ mice lack eosinophils and IL-4R^−/−^ and IL-4R^−/−^/IL-5^−/−^ mice lack alternatively activated macrophages. Total number of thoracic cavity cells in IL-4R^−/−^, dblGATA, IL-5^−/−^, IL-4R^−/−^/IL-5^−/−^ mice and wildtype (WT) controls at 71 (**a**) and 119 days (**b**) post-*L. sigmodontis*-infection. Total thoracic cavity macrophage numbers (**c**, **d**), RELMα positive alternatively activated macrophage (AAM, **e**, **f**) and eosinophil numbers (**g**, **h**) at 71 and 119 days post-*L. sigmodontis*-infection (**a**–**h**), as well as expression of CD54 (**i**) and CD69 on eosinophils (**j**) at 71 days post-*L. sigmodontis*-infection and naïve WT controls (**g**–**j**). Results are shown as medians. Data were analyzed using one-way ANOVA followed by Dunnett’s test (**c**, **g**) and Kruskal-Wallis followed by Dunn’s multiple comparison test (**a**, **b**, **d**–**f**, **h**–**j**). *P < 0.05, **P < 0.01, ***P < 0.001. Data shown in **a**, **c**, **e**, **g** are pooled from two independent experiments at 71 dpi with 10–16 mice per group. Remaining panels (**b**, **d**, **f**, **h**) are from one experiment at 119 dpi with 6–10 mice per group. **i**, **j** Representative of one independent experiment at 71 dpi with 4–6 mice per group

In order to investigate whether the pronounced number of MF and adult worms in immunodeficient mice were associated with different frequencies of immune cell populations, flow cytometric analyses were performed on thoracic cavity and spleen cells of WT, IL-4R^**−/−**^, dblGATA, IL-5^**−/−**^, and IL-4R^**−/−**^/IL-5^**−/−**^ mice at 71 and 119 dpi. Thoracic cavity cell counts were lower in dblGATA, IL-5^**−/−**^ and IL-4R^**−/−**^/IL-5^**−/−**^ mice in comparison to WT controls at 71 dpi, reaching statistical significance for IL-5^**−/−**^ and IL-4R^**−/−**^/IL-5^**−/−**^ mice (Fig. 2a). Analysis at 119 dpi did not show any significant differences in the thoracic cavity cell numbers among the tested mouse strains (Fig. 2b). At 71 and 119 dpi the absence of IL-4R led to reduced absolute numbers of macrophages (Fig. 2c, d) and a lack of AAMs (Fig. 2e, f) within the thoracic cavity of IL-4R^**−/−**^ and IL-4R^**−/−**^/IL-5^**−/−**^ mice. In contrast, the total numbers of AAMs tended to be increased in IL-5^**−/−**^ and were significantly increased in dblGATA mice at 119 dpi, which was associated with the increased adult worm burden at that time point. As expected, naïve WT animals had significantly lower numbers of eosinophils and IL-5^**−/−**^ as well as dblGATA mice had decreased numbers of eosinophils in comparison to infected WT controls (Fig. 2g, h). Less total eosinophils counts were also observed in IL-4R^**−/−**^ and IL-4R^**−/−**^/IL-5^**−/−**^ animals. Furthermore, eosinophil activation as indicated by expression of CD54 /ICAM-1 (Fig. 2i) and CD69 (Fig. 2j) were significantly increased upon *L. sigmodontis* infection in WT animals and reduced in infected dblGATA mice compared to infected WT animals.

**Fig. 3:**
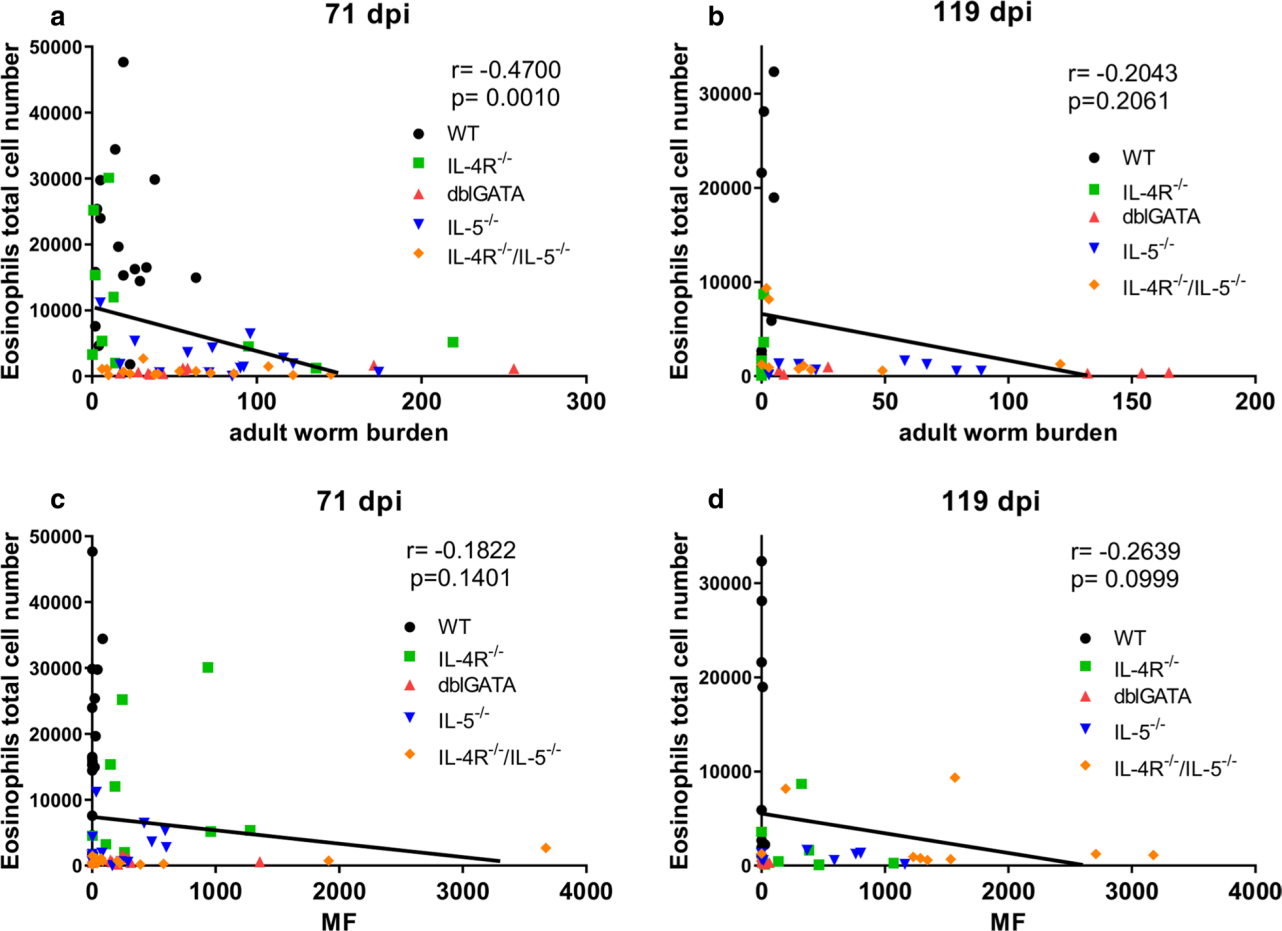
Negative correlation of thoracic cavity eosinophil numbers and adult worm burden. Spearman correlation of the total thoracic cavity eosinophil numbers and adult worm burden (**a**, **b**) and peripheral blood MF counts (**c**, **d**) at 71 (**a**, **c**) and 119 days (**b**, **d**) after *L. sigmodontis*-infection in IL-4R^−/−^ (green dots), dblGATA (red dots), IL-5^−/−^ (blue dots), IL-4R^−/−^/IL-5^−/−^ (orange dots) mice and wildtype controls (black dots). Data shown in **a**, **c** are pooled from two independent experiments at 71 dpi with 10–16 mice per group. Remaining panels (**b**, **d**) are from one experiment at 119 dpi with 6–10 mice per group

**Fig. 4:**
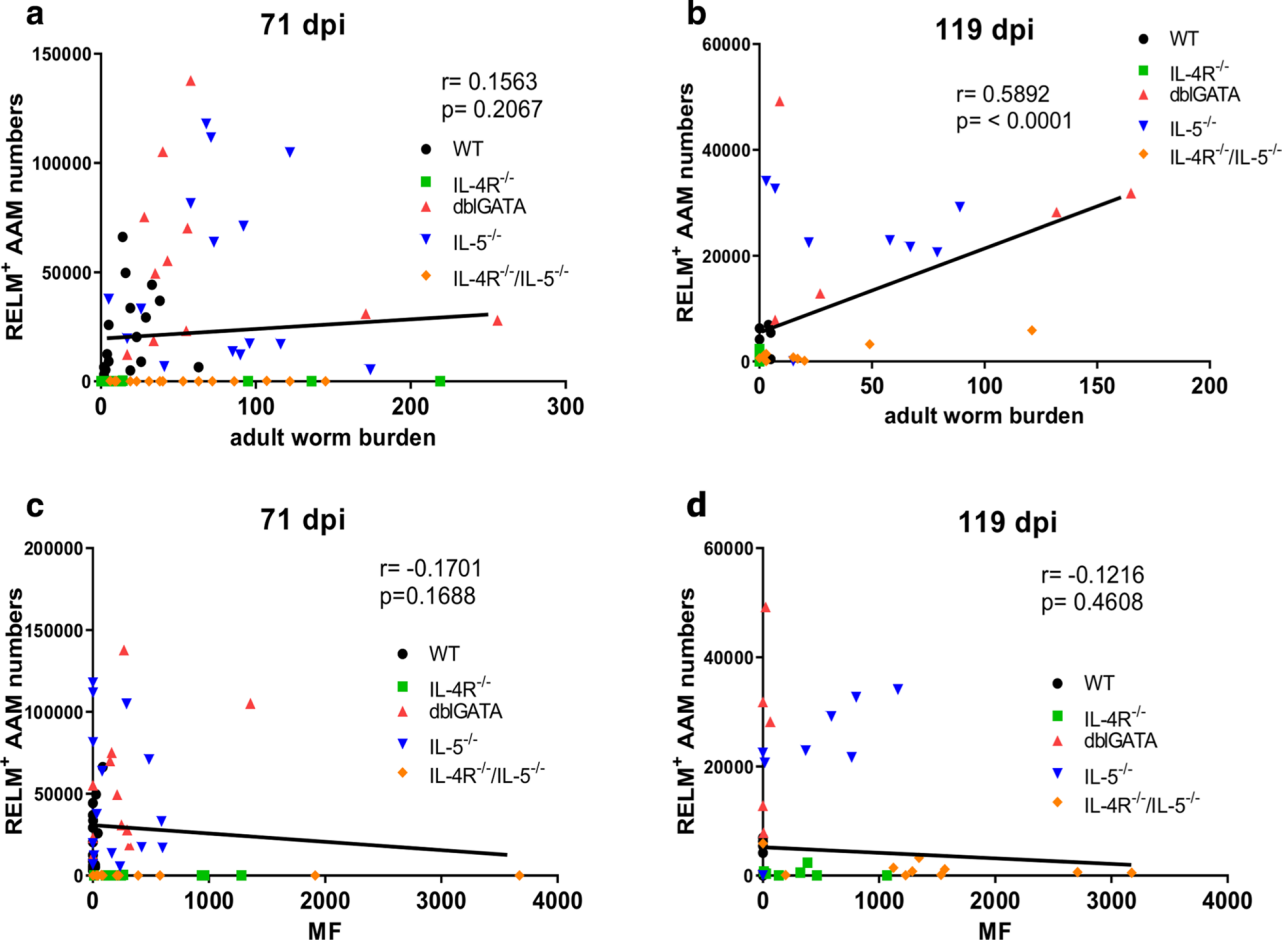
Contrasting correlation of thoracic cavity alternatively activated macrophage numbers and adult worm burden and MF counts. Spearman correlation of the total thoracic cavity RELMα-positive macrophage numbers and adult worm burden (**a**, **b**) and peripheral blood microfilariae (MF) counts (**c**, **d**) at 71 (**a**, **c**) and 119 days (**b**, **d**) post-*L. sigmodontis*-infection in IL-4R^−/−^ (green dots), dblGATA (red dots), IL-5^−/−^ (blue dots), IL-4R^−/−^/IL-5^−/−^ (orange dots) mice and wildtype controls (black dots). Data shown in **a**, **c** are pooled from two independent experiments at 71 dpi with 10–16 mice per group. Remaining panels (**b**, **d**) are from one experiment at 119 dpi with 6–10 mice per group

A negative correlation was observed for thoracic cavity eosinophil numbers and adult worm counts at 71 dpi (Correlation: *r*_(67)_ = − 0.47, *P* = 0.001; Fig. 3a), which vanished at 119 dpi (Fig. 3b). Thoracic cavity eosinophil counts were negligible negatively correlated to the MF load at 71 (Correlation: *r*_(67)_ = − 0.18, *P* = 0.14) and at 119 dpi (Correlation: *r*_(41)_ = − 0.26, *P* = 0.10) (Fig. 3c, d). Thoracic cavity AAM counts on the other hand did not correlate with adult worm counts at 71 dpi (Fig. 4a), but positively correlated (moderate) at 119 dpi (Correlation: *r*_(41)_ = 0.59, *P* = 0.0001; Fig. 4b). AAM numbers showed a negligible negative correlation with the MF load at 71 dpi (Correlation: *r*_(69)_ = − 0.17, *P* = 0.17; Fig. 4c) and at 119 dpi (Correlation: *r*_(41)_ = − 0.12, *P* = 0.46; Fig. 4d). The results indicate that thoracic cavity eosinophils impair adult worm survival and reduce MF levels, whereas AAMs have a low impact on MF counts.

**Fig. 5:**
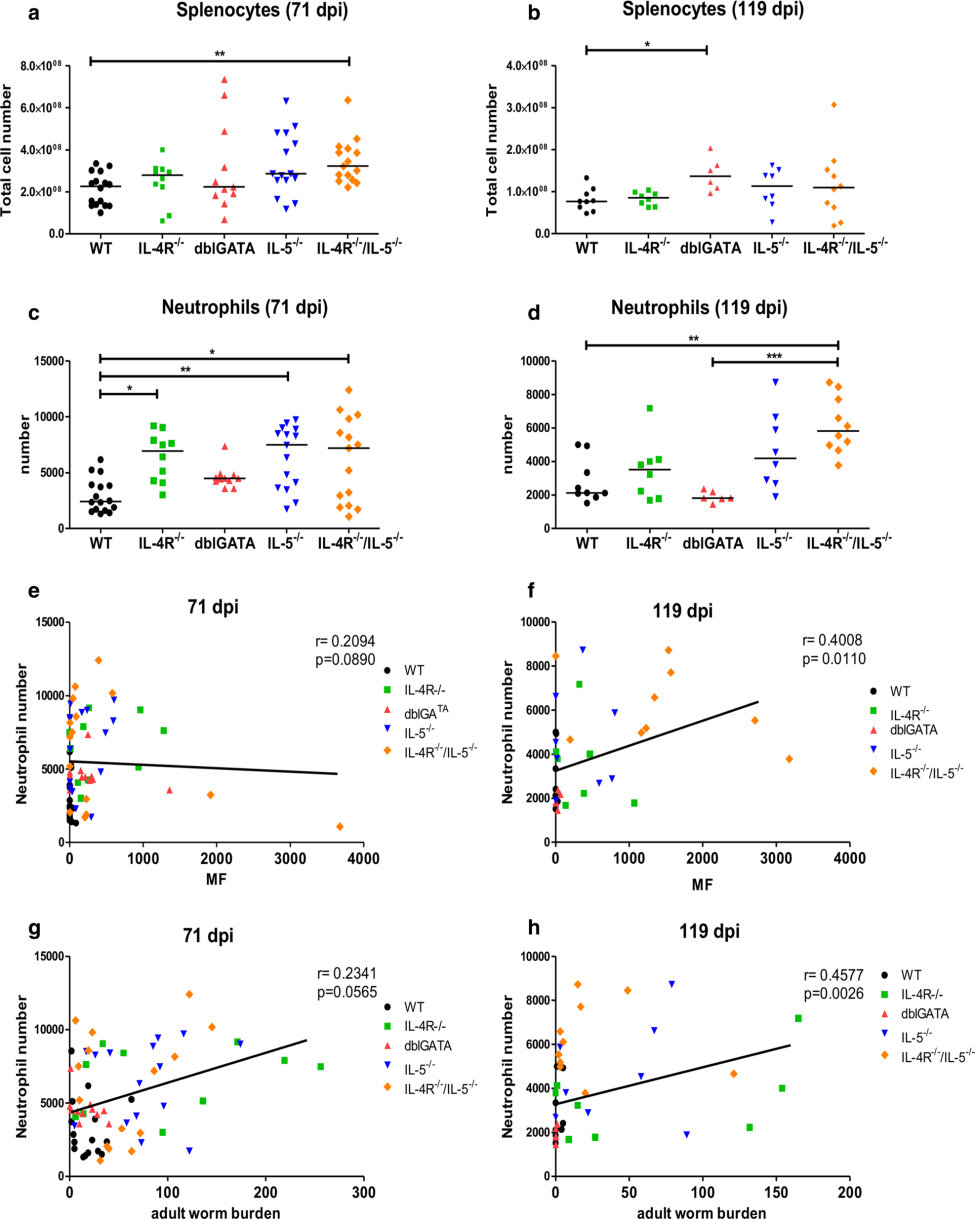
Neutrophil numbers in the spleen correlate positively with the adult worm burden and microfilariae load. Total number of spleen cells (**a**, **b**) and neutrophils (**c**, **d**) in IL-4R^−/−^, dblGATA, IL-5^−/−^, IL-4R^−/−^/IL-5^−/−^ mice and wildtype (WT) controls 71 and 119 days post-*L. sigmodontis*-infection. Spearman correlation of the total neutrophil spleen cell numbers and peripheral blood microfilariae (MF) counts (**e**, **f**) and adult worm burden (**g**, **h**) at 71 and 119 days post-*L. sigmodontis*-infection in IL-4R^−/−^ (green dots), dblGATA (red dots), IL-5^−/−^ (blue dots), IL-4R^−/−^/IL-5^−/−^ (orange dots) mice and wildtype controls (black dots). Results are shown as medians (**a**–**d**) and Spearman correlation (**e**–**h**). Differences were analyzed for statistical significance using the one-way ANOVA followed by Dunnett’s test (**b**–**d**) and Kruskal–Wallis test followed by Dunn’s multiple comparison test (**a**). *P < 0.05, **P < 0.01, ***P < 0.001. Data in **a**, **c**, **e**, **g** are pooled from two independent experiments at 71 dpi with 10–16 mice per group. Remaining panels (**b**, **d**, **f**, **h**) are from one experiment at 119 dpi with 6–10 mice per group

With regard to immunological changes within the spleen, spleen cell numbers were significantly increased for IL-4R^**−/−**^/IL-5^**−/−**^ mice in comparison to WT controls at 71 dpi (Fig. 5a). At 119 dpi, spleen cell numbers were comparable for all tested mouse strains, although a statistically significant increase was present for dblGATA mice in comparison to WT controls (Fig. 5b). Absolute numbers of CD4+ T cells, CD8+ T cells, neutrophils, macrophages and eosinophils were investigated within the spleen on 71 and 119 dpi (Fig. 5, Additional file 3: Figure S2). CD4+ T cell total numbers showed a significant decrease for IL-4R^**−/−**^, IL-5^**−/−**^ and IL-4R^**−/−**^/IL-5^**−/−**^ mice at 71 dpi (Additional file 3: Figure S2a), whereas CD4+ T cells were significantly lower in dblGATA and IL-5^**−/−**^ mice compared to IL-4R^**−/−**^ mice at 119 dpi (Additional file 3: Figure S2b). CD8+ T cell numbers were significantly decreased in IL-4R^**−/−**^, IL-5^**−/−**^ and IL-4R^**−/−**^/IL-5^**−/−**^ on 71 dpi (Additional file 3: Figure S2c) and continued being decreased on 119 dpi for IL-4R^**−/−**^ and IL-4R^**−/−**^/IL-5^**−/−**^ deficient mice compared to WT controls (Additional file 3: Figure S2d). Total numbers of macrophages at 71 dpi showed a significant increase in IL-4R^**−/−**^/IL-5^**−/−**^ compared to WT and IL-4R^**−/−**^ mice (Additional file 3: Figure S2e). At 119 dpi no differences were observed in regard to macrophage numbers (Additional file 3: Figure S2f). Eosinophils at 71 dpi (Additional file 3: Figure S2g) did show a statistically significant increase in IL-4R^**−/−**^ mice compared to WT controls and eosinophil numbers on 119 dpi were significantly decreased in dblGATA and IL-5^**−/−**^ animals compared to WT mice (Additional file 3: Figure S2h). Eosinophil counts in the spleen did hereby not significantly correlate with the MF load at 71 and 119 dpi (Additional file 3: Figure S2i, j), but correlated (low) negatively with the adult worm counts at 119 dpi (*r*_(41)_ = − 0.267, *P* = 0.100; Additional file 3: Figure S2k, l). The number of neutrophils was significantly increased in spleens of IL-4R^**−/−**^, IL-5^**−/−**^ and IL-4R^**−/−**^/IL-5^**−/−**^ mice at 71 dpi (Fig. 5c) and in IL-4R^**−/−**^/IL-5^**−/−**^ mice at 119 dpi compared to WT controls (Fig. 5d). A low positive correlation was observed for neutrophil counts in the spleen with the MF load (71 dpi: *r*_(68)_ = 0.209, *P* = 0.089; 119 dpi: *r*_(39)_ = 0.400, *P* = 0.011; Fig. 5e, f) as well as the adult worm burden at 119 dpi (71 dpi: *r*_(67)_ = 0.234, *P* = 0.057; 119 dpi: *r*_(41)_ = 0.458; *P* = 0.003; Fig. 5g, h).

### Thoracic cavity cytokine concentrations do not correlate with microfilariae and adult worm burden

**Fig. 6:**
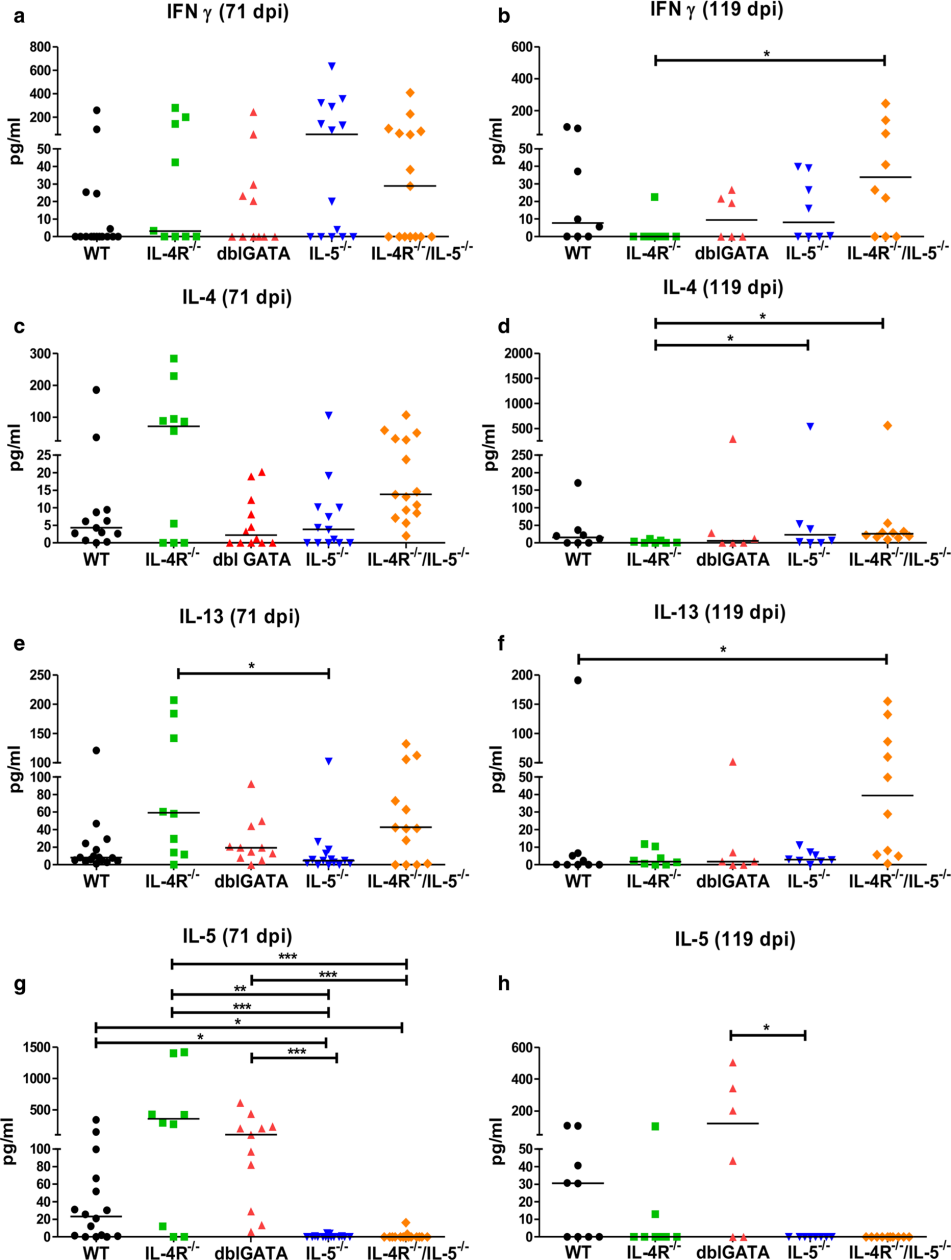
Impact of the lack of IL-5, dblGATA, IL-4R and IL-4R/IL-5 on the thoracic cavity cytokine milieu in *L. sigmodontis*-infected mice. Cytokine concentrations of IFNγ (**a**, **b**), IL-4 (**c**, **d**), IL-13 (**e**, **f**) and IL-5 (**g**, **h**) in the thoracic cavity lavage 71 and 119 days post-*L. sigmodontis*-infection of wildtype (WT), IL-4R^−/−^, dblGATA, IL-5^−/−^ and IL-4R/IL-5^−/−^ mice. Results are shown as medians. Differences were analyzed for statistical significance using the Kruskal–Wallis test followed by Dunn’s multiple comparison test. *P < 0.05, **P < 0.01, ***P < 0.001. Data shown in **a**, **c**, **e**, **g** are pooled from two independent experiments at 71 dpi with 10–16 mice per group. Remaining panels (**b**, **d**, **f**, **h**) are from one experiment at 119 dpi with 6–10 mice per group

In order to investigate whether microfilaremia and adult worm burden correlate with changes in the local cytokine milieu, we quantified cytokines within the thoracic cavity lavage, namely the Th1 cytokine IFNγ, as well as the type 2 cytokines IL-4, IL-5 and IL-13 (Fig. 6). IFNγ production did not differ among the different groups at 71 dpi (Fig. 6a) but was significantly elevated in IL-4R^−/−^/IL-5^−/−^ mice at 119 dpi compared to IL-4R^−/−^ mice (Fig. 6b). Of note, IL-4 (Fig. 6c, d) and IL-13 (Fig. 6e, f) levels were increased in IL-4R^−/−^ and IL-4R^−/−^/IL-5^−/−^ compared to WT animals at 71 dpi, suggesting either an elevated IL-4 and IL-13 production due to a missing negative feedback loop in IL-4R^−/−^ animals or a binding of IL-4 to the IL-4R of mice expressing the receptor. In contrast, IL-5 cytokine levels were absent in IL-4R^−/−^/IL-5^−/−^ and IL-5^−/−^ mice (Fig. 6g, h). Th1 and Th2 cytokine levels did neither correlate with adult worm burden nor MF load; correlations are summarized in Table 1.

**Table 1:** IL-4, IL-5, IL-13, and IFNγ thoracic cavity concentrations do not correlate with the MF load or adult worm burden. Spearman correlation of IL-4, IL-5, IL-13, and IFNγ cytokine levels within the thoracic cavity lavage at 71 and 119 days post-*L. sigmodontis* infection with the total worm numbers and MF load

Cytokine	71 dpi		119 dpi	
	*r*	*P*	*r*	*P*
Microfilariae				
IL-4	0.34	0.04	− 0.04	0.79
IL-5	0.10	0.62	− 0.33	0.13
IL-13	0.18	0.26	0.36	0.02
IFNy	0.03	0.84	0.16	0.34
Worm burden				
IL-4	0.01	0.95	0.06	0.73
IL-5	0.19	0.35	− 0.34	0.11
IL-13	0.01	0.93	0.19	0.23
IFNy	0.07	0.65	− 0.04	0.81

## Discussion

In the present study we directly compared the impact of IL-4R, IL-5, eosinophils (dblGATA) and both IL-4R + IL-5 on the infection with the filarial nematode *L. sigmodontis*. As IL-4R is required for the induction of AAM and IL-5 for the generation and maintenance of eosinophils, we further correlated our parasitological results with these two cell types.

A previous study using *L. sigmodontis* showed that the lack of IL-4, IL-4R, or IL-5 leads to an increased and extended microfilaremia in BALB/c mice and that the lack of IL-5 facilitates adult worm survival [36]. It is further known that *L. sigmodontis* infection triggers an eosinophilia which helps to eliminate the adult worms [32, 35]. Therefore, our results showing an increased microfilaremia in IL-4R^−/−^, IL-5^−/−^, IL-4R^−/−^/IL-5^−/−^ and dblGATA mice in comparison to WT animals are in accordance with these previous studies. Furthermore, our study demonstrates that IL-4R^−/−^/IL-5^−/−^ mice had significantly more embryonal stages compared to WT and IL-5^−/−^ mice, indicating that the MF release is increased in this mouse strain. The direct comparison of these immunodeficient mice in our study further highlights that the lack of IL-5 and eosinophils rather than IL-4R extends the microfilaremia and that there is a cumulative effect with a combined lack of both IL-5 and IL-4R, resulting in the highest MF load over time. The increased adult worm burden at a late time point of infection in animals lacking eosinophilia (IL-5^−/−^, IL-4R^−/−^/IL-5^−/−^, dblGATA) further suggests that the extended microfilaremia is rather due to this prolonged adult worm survival than an impaired MF clearance. Accordingly, Volkmann et al. [36] associated in their study an extended adult worm survival with a prolonged microfilaremia and showed that the survival of injected MF was comparable in WT, IL-4^−/−^ and IL-4R^−/−^ mice and was only slightly extended in IL-5^−/−^. Eosinophil deficient PHIL mice on the other hand had an impaired clearance of injected *Brugia malayi* MF [42], indicating that eosinophils contribute to some extend to the *in vivo* clearance of MF. *In vivo* clearance of MF is supported by the spleen and reduced spleen mass was shown to facilitate MF survival [41, 43], whereas the tested mouse strains in our study had all enlarged spleens in comparison to WT controls. *In vitro* studies showed that eosinophils as well as neutrophils adhere to MF and inhibit MF motility and survival [44–47], a process which is mediated by the eosinophil granula proteins EPO (eosinophil peroxidase), MBP (major basic protein), ECP (eosinophilic cationic protein), EDN (eosinophil-derived neurotoxin) and in part by extracellular DNA traps [46, 48, 49], suggesting that eosinophils may directly impact MF survival *in vivo*.

Our study further demonstrates that at 71 dpi the onset of microfilaremia is accelerated in the absence of IL-4R, as was shown in IL-4R^−/−^ and IL-4R^−/−^/IL-5^−/−^ mice, and to a lesser degree in eosinophil-deficient dblGATA and IL-5^−/−^ mice. This advanced onset of microfilaremia in the tested immune-deficient mice was further associated with increased female worm lengths at 71 dpi but not 119 dpi, suggesting that the filarial development is faster in the absence of IL-4R, IL-5 and eosinophils. These findings are in contrast to a study demonstrating that in the absence of IL-5 or eosinophils *L. sigmodontis* larvae have a delayed molting into the L4 stage and that co-administration of recombinant IL-5 with L3 inoculation leads to an earlier onset of microfilaremia and a higher microfilariae load [50]. The discrepancy of this study and ours may be caused by the host background, as we used susceptible BALB/c mice, whereas the Babayan study used for some experiments semi-resistant C57BL/6 mice and concentrated on earlier time points post-infection as we did. Furthermore, the continuous lack of eosinophils in dblGATA and IL-5^−/−^ mice in our study contrasted with the time restricted exposure to IL-5 during L3 inoculation in the study of Babayan et al. [50]. Future studies should therefore investigate to which degree host background and exposure time to eosinophils/IL-5 impact *L. sigmodontis* development. Lack of eosinophils (dblGATA), IL-4R and IL-4R/IL-5 enabled the development of microfilaremia in 100% of the tested animals of our study, which is in-line with the enhanced embryogenesis in IL-4R^−/−^/IL-5^−/−^ mice. In contrast, IL-5^−/−^ mice showed no such increased patency in comparison to WT controls, suggesting that the phenotypes of IL-5^−/−^ mice and dblGATA mice differ to some degree. Correlations of thoracic cavity eosinophil numbers further revealed that eosinophils are negatively correlated with the adult worm burden at 71 dpi, supporting the essential role of eosinophils in protective immune responses against filariae [32, 35].

Expansion of AAM in the thoracic cavity of *L. sigmodontis-*infected mice is dependent on the IL-4R [21] and the absence of AAM in IL-4R^−/−^ and IL-4R^−/−^/IL-5^−/−^ mice was associated with reduced total numbers of thoracic cavity macrophages. Interestingly, a positive correlation was observed between total AAM cell counts and worm burden at 119 dpi. While AAM are known suppressors and mediators of helminth-induced immune modulation [22, 51–53], it remains to be confirmed, whether the observed positive correlation in our study is due to the known expansion of AAM during helminth infection [21] or whether AAM facilitate adult worm survival. RELMα, one of the molecules produced by AAM, inhibits Th2-associated inflammatory responses during helminth infection [52] and impairs helminth expulsion [54], indicating that AAM-derived factors can improve helminth survival. On the other hand, AAM have been shown to drive eosinophilia in *B. malayi* infected mice and depletion of AAM suppressed eosinophilia and filarial clearance [55]. Lack of AAMs were associated in our study with decreased eosinophil counts after 71 dpi (*P *> 0.05) and 119 dpi (*P *< 0.05). Furthermore, at 119 dpi, mainly animals lacking eosinophils (IL-4R^−/−^/IL-5^−/−^, dblGATA and IL-5^−/−^ mice) had remaining adult worms, which was probably attributed to the impaired eosinophil-mediated clearance of adult worms. Thus, the positive correlation between the adult worm burden and the numbers of AAM at 119 dpi in our study may not be due to a beneficial effect of AAM, but rather due to the lack of eosinophils, allowing the maintenance of adult worms and AAM.

Thoracic cavity cytokine levels of IL-4, IL-13 and IL-5 neither correlated with the adult worm burden nor microfilaremia, although outliers with highest IL-4 and IFNγ cytokine levels had the highest adult worm burden and thoracic cavity IFNy levels tended to be increased in all tested immune-deficient mice.

An induction of IFNγ responses was previously observed by injections of MF into naïve animals, suggesting that MF trigger type 1 immune responses [56]. Increased IFNγ levels may therefore contribute to the removal of adult worms, as IFNγ-deficient mice have an impaired neutrophil-mediated clearance of *L. sigmodontis* adult worms and an increased microfilaremia [28]. The present study showed that the expansion of the neutrophil population within the spleen correlated positively with the MF load and the adult worm burden.

## Conclusions

Our results indicate that the combined lack of IL-4R and eosinophils in IL-4R^−/−^/IL-5^−/−^ mice leads to a further increased susceptibility for *L. sigmodontis* infection, which included an enhanced embryogenesis, an earlier onset of microfilaremia, development of microfilaremia in 100% of the infected animals with highest MF loads, and an extended adult worm survival. Therefore, IL-4R^−/−^/IL-5^−/−^ signalling may also be essential for the control of infections with human pathogenic filariae. Accordingly, the human pathogenic filaria *Loa loa* was shown to survive up to 70 days after inoculation with the infective larval stage in IL-4R^−/−^/IL-5^−/−^ mice and developed into immature adult worms [57]. Furthermore, IL-4R^−/−^/IL-5^−/−^ mice may be interesting for the screening of potential drug candidates to assess their macrofilaricidal efficacy. Extended adult worm survival and increased adult worm burden in IL-4R^−/−^/IL-5^−/−^ mice allows a longer time between treatment start and analysis of the adult worm burden, which facilitates the identification of active compounds with a slower macrofilaricidal efficacy. As all IL-4R^−/−^/IL-5^−/−^ mice develop microfilaremia, compounds can be also tested for their microfilaricidal efficacy by administering drug candidates during patency, thereby circumventing the need for fully susceptible jirds, which are more laborious to maintain. The lack of AAM and eosinophils in IL-4R^−/−^/IL-5^−/−^ mice further allows to determine whether the efficacy of drug candidates depends on these cell types or an intact host immune system. Therefore, the increased susceptibility of IL-4R^−/−^/IL-5^−/−^ mice for filarial infections may provide new paths in filarial research.

## Additional files

**Additional file 1: Figure S1.** Representative gating strategy using thoracic cavity cells of a wildtype mouse 71 dpi. Lymphocytes (P3) were assessed for their expression of CD4 and CD8 to identify CD4+ and CD8+ T cells. All cells (P1) were analysed to differentiate eosinophils (SiglecF^+^ F4/80^-^) and macrophages (SiglecF^low/int^ F4/80^+^). SiglecF^-^ F4/80^-^ cells (P2) were analyzed for their expression of Gr-1 to identify neutrophils. Gating of RELMα^+^ macrophages was performed using the fluorescence minus one (FMO) approach.

**Additional file 2: Table S1.** Mean adult worm burden and microfilariae (MF) counts per 50 µl of blood and standard deviation (SD) as well as minimum and maximum adult worm/microfilariae counts are shown as well as the frequency of animals developing microfilaremia. Statistical significance was determined by Mann-Whitney U-test.

**Additional file 3: Figure S2.** Total spleen cell CD4+ T cell (**a**, **b**), CD8+ T cell (**c**, **d**), macrophage (**e**, **f**) and eosinophil numbers (**g**, **h**) in IL-4R^−/−^, dblGATA, IL-5^−/−^, IL-4R^−/−^/IL-5^−/−^ mice and wildtype (WT) controls at 71 and 119 dpi. Spearman correlation of the total eosinophil spleen cell numbers and peripheral blood microfilariae (**i**, **j**) and adult worm counts (**k**, **l**) at 71 and 119 dpi. Differences were analyzed for statistical significance using the one-way ANOVA followed by the Dunnett’s test (**a**, **d**) and Kruskal-Wallis test followed by the Dunn’s multiple comparison test (**b**, **c**, **e-h**). *P < 0.05, **P < 0.01, ***P < 0.001. Data in **c**, **e**, **g**, **i** and **k** are pooled from two independent experiments at 71 dpi with 10–16 mice per group; data shown in **a** are a representative of two independent experiments with 5–10 mice per group, as it did not pass the test for homodesticity. Remaining figures (**b**, **d**, **f**, **h**, **j**, **l**) are from one experiment at 119 dpi with 6–10 mice per group.

## Data Availability

All data generated or analyzed during the present study are included in this published article and its additional files.

## Abbreviations

APC: allophycocyanin
AAM: alternatively activated macrophages
BSA: bovine serum albumin
CD: cluster of differentiation
DPI: days post-infection
EPO: eosinophil peroxidase
F4/80: mouse epidermal growth factor-like module-containing mucin-like hormone receptor-like 1
FITC: fluorescein isothiocyanate
IL: interleukin
IL-4R: IL-4 receptor
IFNγ: interferon gamma
Ig: immunglobulin
MBP: major basic protein
MF: microfilariae
PE: phycoerythrin
RBC: red blood cell
RELMα: resistin-like molecule alpha
RPMI: Roswell Park Memorial Institute
Siglec F: sialic acid-binding immunoglobulin superfamily lectins
ILC2s: type 2 innate lymphoid cells
WT: wildtype
PBS: phosphate-buffered saline
