# Supplementary material for: Susceptibility to L. sigmodontis infection is highest in animals lacking IL-4R/IL-5 compared to single knockouts of IL-4R, IL-5 or eosinophils
Source: Parasit Vectors. 2019 May 20;12:248. doi: 10.1186/s13071-019-3502-z (PMC6528299; doi:10.1186/s13071-019-3502-z)
Supplement: Supplementary file 2 — Additional file 2: Table S1. Mean adult worm burden and microfilariae (MF) counts per 50 µl of blood and standard deviation (SD) as well as minimum and maximum adult worm/microfilariae counts are shown as well as the frequency of animals developing microfilaremia. Statistical significance was determined by Mann-Whitney U-test. [file 13071_2019_3502_MOESM2_ESM.docx]

**Additional file 2: Table S1. Eosinophil-deficient dblGATA mice have an increased adult worm burden and microfilaremia.** Mean adult worm burden and microfilariae counts per 50µl and standard deviation as well as minimum and maximum adult worm counts/microfilariae are shown as well as the frequency of animals developing microfilaremia. Statistical significance was determined by Mann-Whitney U-test.

| **Microfilariae** | | **MEAN** | **SD** | **Min-Max** | **% MF positive** | **Significance** |
| --- | --- | --- | --- | --- | --- | --- |
| 56 dpi | WT (n=10) dblGATA (n=10) | 4.1 5.6 | 9.3 13.6 | 0-30 0-44 | 50% 60% | ns |
| 63 dpi | WT (n=10) dblGATA (n=10) | 7.6 41.8 | 14.3 73.1 | 0-47 0-209 | 70% 70% | ns |
| 69 dpi | WT (n=10) dblGATA (n=10) | 10.0 81.9 | 21.1 129.8 | 0-68 0-408 | 50% 70% | ns |
| 76 dpi | WT (n=10) dblGATA (n=10) | 9.6 101.4 | 24.3 174.0 | 0-78 0-561 | 40% 70% | p < 0.05 |
| **Worm burden** | | **MEAN** | **SD** | **Min-Max** | **% worm positive** | **Significance** |
| 30 dpi | WT (n=7) dblGATA (n=5) | 5.0 12.2 | 4.4 7.3 | 1-12 3-22 | 100% 100% | ns |
| 60 dpi | WT (n=5) dblGATA (n=6) | 4.8 12.5 | 3.7 5.2 | 0-9 7-19 | 80% 100% | p < 0.05 |
| 90 dpi | WT (n=18) dblGATA (n=18) | 1.9 10.7 | 3.2 13.0 | 0-12 0- 51 | 50% 83% | p < 0.01 |
